# Explaining Symptoms in Systemic Therapy. Does Triadic Thinking Come Into Play?

**DOI:** 10.3389/fpsyg.2020.00597

**Published:** 2020-05-05

**Authors:** Valeria Ugazio, Roberto Pennacchio, Lisa Fellin, Stella Guarnieri, Pasquale Anselmi

**Affiliations:** ^1^European Institute of Systemic-relational Therapies, Milan, Italy; ^2^Department of Human and Social Sciences, University of Bergamo, Bergamo, Italy; ^3^Scarpellini Family Counselling Centre, Bergamo, Italy; ^4^School of Psychology, University of East London, London, United Kingdom; ^5^Department of Philosophy, Sociology, Education, and Applied Psychology, University of Padua, Padua, Italy

**Keywords:** triadic thinking, causal explanations, symptoms, mental disorders, therapeutic change, systemic psychotherapy, psychopathology, attributions

## Abstract

The main aim of this study is to explore the breadth of the inference field and the type of etiopathogenetic contents of symptom explanations provided by the client and therapist in the first two psychotherapy sessions conducted using a systemic approach. Does the therapist use triadic explanations of psychopathology as suggested by her approach? And do clients resort almost exclusively to monadic and dyadic explanations as did the university students in our previous study? What kind of explanations do they propose? The coding system “1 to 3: from the monad to the triad” was applied to the transcripts of 25 individual systemic therapies conducted by the same therapist. This manual allows coding of the inference field of symptom explanations according to three categories: monadic, dyadic, and triadic. These three broad categories are also used to analyze the etiopathogenetic content of each explanation: traumatic, intrapersonal, and interpersonal. Our findings showed that clients and their therapist actually used different inference fields: clients resorted almost exclusively to monadic and dyadic explanations, whereas their therapist included the triadic explanatory level. Moreover, the therapist provided more interpersonal explanations than her clients. Hence, the dissonance between client and therapist about the inference fields – a crucial premise of one of the most accepted ideas of therapeutic change according to systemic therapies – is proven, at least among our participants. Thanks to this dissonance, clients and therapists can create a new story, potentially able to change clients’ feelings, without disconfirming their emotions.

## Introduction

The context for systemic therapies is the matrix of meaning. As claimed by Watzlawick et al. (1967) in *Pragmatics of Human Communication*, perhaps the systemic approach’s best-known text, “a phenomenon remains inexplicable as long as the range of observation is not wide enough to include the context in which the phenomenon occurs” (Watzlawick et al., 1967, pp. 20–21). But how broadly should the field of observation be widened? Until now, systemic therapies have no detailed indication in regard to this, but they do foster the idea that the field of observation should be broadened to at least triadic contexts and, consequently, the development of triadic heuristics.

One of the specificities that still distinguishes systemic therapists from colleagues with other approaches is the use of at least triadic schemes for the explanation of symptoms and related dysfunctional behaviors (Flaskas, 2012). Almost all pioneers of systemic and family therapies placed the triad at the base of their theories and practices. Haley (1969), claimed that the triad was the minimum unit of observation within the systemic approach. Before that, Weakland (1960) had provided a triadic interpretation of the *double bind* (Bateson et al., 1956), a core concept of the emerging systemic therapies. Triangles and triangulations were also at the heart of the clinical developments of many family therapists, including Zuk (1969), Bowen (1966, 1978), Minuchin (Minuchin et al., 1967; Minuchin, 1974), and Selvini Palazzoli et al. (1978, 1980).

During the sixties and the seventies, systemic therapies interpreted the shift of attention from the individual to the triad, or to broader units, as broadening of the observation field. Back then, systemic therapies were mainly family therapies, and the focus was addressed to the here-and-now interactions. Later, the abandoning of the concept of the mind as a black box, the resumption of Batson’s original idea of the contextual mind, the adoption of constructivist paradigms, and the spread of systemic therapeutic practices addressed to individuals gradually changed this methodological choice. The broadening of the observation field is now seen as the broadening of the inference field, that is, the context taken mentally into account by whoever formulates the inference. According to this perspective, broadening the context in which an event occurs is primarily a mental operation of contextualization; it does not necessarily require the observation of a triad, or a broader unit, in interaction. Such a triad, or broader unit, can be not only observed but also evoked. In this perspective, systemic therapies are now characterized by the use of triadic inferential processes and by the inclusion of methods able to elicit them, differentiating themselves from other psychotherapeutic models which are characterized by monadic heuristics, based on individual and intrapsychic processes, or dyadic heuristics, based on the individual relating with a significant other.

Due to this methodological transformation, systemic therapies are no longer identified with family therapy and have increasingly distinguished themselves as a way of thinking that can be used in different therapeutic settings (Heatherington et al., 2015). The choice of who to include in therapy – individuals, couples, families, or siblings – has become a technical decision, which changes the therapeutic strategy but not the way of thinking guiding therapy (Boscolo and Bertrando, 1996). Also, in individual therapies, the therapist envisions the patient and the therapeutic relationship as part of a broader system that includes the family contexts of which the client is part, including those not present during the session. And even in an individual session, one of the most characteristic interventions for a systemic therapist when facing disruptive emotions, symptomatic behaviors, or lacerating conflicts is to contextualize them in an interactional matrix, involving at least three people.

A brief clinical vignette, taken from the participants of this study, allows us to clarify the concept of the monadic, dyadic, and triadic inference fields.^1^ Sonia, a 30-year-old bulimic patient, since adolescence, used to go from size 8 to 16 in only 2 months. She suffered from a devastating hatred for her mother, who, she blamed for her eating disorder and for all the emotional difficulties which had troubled her life. Due to this fierce conflict, Sonia refused to involve her family in therapy, at least at the beginning. “When I was sixteen, I wanted to throw boiling oil in my mother’s face and I used to smash doors, and then I would run and vomit,” Sonia remembered. Since then, many things had changed, but Sonia’s hostility toward her mother had remained unaltered, and the belief that her mother was responsible for her disorder had taken root. “My mother is the cause of my eating disorder; I have understood it through years of therapy (…). Once I thought I was in the wrong, because I am a coward, I don’t have the courage to face situations,” added Sonia, who provided us with a clear example of dyadic explanation of her pathology (“my mother is the cause of my eating disorder”) and also an equally good example of monadic explanation (“I thought I was in the wrong, because I am coward”). Sonia described herself as the opposite of her mother. The mother was depicted as a force of nature, a goddess of war, flashy, and full of energy. “She is awfully self-confident, she always has the courage to express what she has in mind,” Sonia admitted. The daughter, of a sober and shy beauty, constructed herself as controlled and refined, in contrast to her mother. She spoke in a low voice and dressed understatedly.

But why did Sonia hate her mother so badly? She was described as imperious, flamboyant, but she had always favored Sonia over her brothers in the choice of schooling, sports, and shopping, as the daughter admitted. And how was it that the father and brothers, who should have had many more reasons for resentment toward her mother, according to what Sonia reported to the therapist, did not seem to be gripped by resentment?

This more complex plot, which a systemic therapist usually constructs when faced with a symptom or a symptomatic behavior, opens up new questions. For example, what role had the father and brothers played in fuelling Sonia’s hatred toward her mother? Did Sonia express a resentment matured in her relationship with her mother, or did she give voice to the resentment of other family members? And why did she take on this task?

These questions are the result of triadic thinking and of an interviewing technique characterized by so-called circular and reflexive questions (Selvini Palazzoli et al., 1980; Tomm, 1987, 1988). Thanks to these techniques, clients, together with their therapist, become active protagonists in the transformation of their individual and family story. In the first place, this triadic thinking is constructed by the clients themselves, through their answers to the therapist’s reflexive and circular questions. Secondly, it allows another story to be told respecting the client’s emotional experience. The therapist does not question Sonia’s hatred. Once she had understood that the young woman was so tightly sealed in a narrative that had always set her against her mother, the therapist tried to explore who and what in the wider context had contributed to the development of Sonia’s unrelenting hatred. The therapist’s questions were directed to bring about a change of *gestalt*, able to modify Sonia’s feelings toward her mother, fuelled by a narrated story that wavers between dyadic explanations, involving only two actors (e.g., “the one responsible is my mother,” “we are too different,” “she has always bothered me physically, even when she tried to get close”) and monadic explanations, focusing only on one person (e.g., “I’m too sensitive,” “I’m a chicken,” “unlike my brother, I’m not able to let things slide on me”).

The hypothesis underlying the therapist’s^2^ questions was that other family members had fed the young woman’s aggressiveness toward her mother. The therapist, according to her model, considered at least three actors. Her field of inference was made up of not only Sonia but also her parents and her brothers, even when they were not present in the therapy. Systemic therapies have in fact always assumed that triadic patterns are an expression of the clients’ lived experience, and therefore are plausible and able to connect the client with silent domains of experience based on emotions. However, empirical research confirming this clinical intuition has long been lacking.

For the past 20 years, thanks to the paradigm of research about mother, father, and child – dubbed Lausanne Triadic Play (LTP) – developed by Fivaz-Depeursinge and colleagues (Fivaz-Depeursinge and Corboz-Warnery, 1999; Fivaz-Depeursinge and Philipp, 2014; McHale et al., 2018), we have had numerous confirmations that our lived story is a triadic one from the beginning of our life. In fact, children, from the first months of life, are able to interact with two partners simultaneously, alternating the gaze between the two parents, and from 9 months onward are capable of complex triadic interactions. These results have been confirmed by longitudinal research in different countries, which leaves no doubt: our lived experience is interwoven with plots that are at least triadic from early infancy.

This is not the case for our narrated story, which seems extraneous to triadic heuristics. The triadic patterns seem extraneous not only to clinical thinking but also to common sense, at least in western cultures. An exception to this is jealousy, which gives rise to three-way games splendidly exemplified in literature and films, such as *Wuthering Heights* (Brontë, 1847). But are there empirical studies able to confirm that common sense is extraneous to triadic thinking?

To the best of our knowledge, only the study run by Ugazio et al. (2012) had explicitly set this goal. It focused on the explanations given by university students to an unexpected enigmatic symptom-like event: a model student communicates his or her decision to leave university shortly before graduation, without giving a reason to his or her parents. This event was presented through four different stimulus situations, corresponding to four different levels of the breadth of the evoked relational context. In the first version, the student was presented alone, without any contextualization^3^ (evoked monadic context); in the second, the student was presented when he or she communicated his or her decision to his or her mother^4^ (evoked dyadic context); and in the third version, his or her decision was communicated to both parents (evoked triadic context).^5^ In the latter, the parents showed opposite emotional responses to their child’s communication^6^ (evoked triadic enigmatic context). The results show that participants made almost exclusive use of monadic and dyadic explanatory schemes. Only in the triadic enigmatic stimulus situation, in which the student received two opposite emotional responses from his or her parents, some explanations emerged in which three actors were involved in a single explanatory scheme.

We therefore have firsthand evidence of how unfamiliar to common sense triadic thinking is also when the stimulus situations evoked contexts involving more actors, as the systemic therapists used to do through their interviewing technique. But what happens when, like in the current study, the event to be explained is a real symptom and it is the actors themselves, the clients, who narrate to their systemic therapist their attempts to give meaning to their symptoms, being actively engaged in the conversation through circular and reflexive questions? Does the field of inference change?

The enigmatic event proposed in the previous study did not put medical culture at stake. When it comes to symptoms, medical culture can encourage a monadic and linear way of interpreting them, especially in this historical period where biological psychiatry has the upper hand (Haslam, 2005; Pescosolido et al., 2010; Kvaale et al., 2013; Lebowitz, 2014; MacDuffie and Strauman, 2017; Lebowitz and Appelbaum, 2019). The students could identify themselves with a colleague who suddenly decided to drop out of university but were not directly involved. The motivation of the participants, in this current study, was incomparably greater than that of the students involved in the study by Ugazio et al. (2012). The participants’ coherence of the self is threatened by the onset of symptoms, and the very voids of meaning opening up in the narrative plot were a powerful stimulus to the participants’ attributive effort. The request for therapy is also a request for meaning. Patient and therapist are therefore motivated to seek reasons for symptoms and other enigmatic behaviors. Precisely because of this joint commitment of the two conversational partners in meaning-making, psychotherapy is one of the natural contexts in which the attributional effort is stronger. This is most true for constructivist and constructionist approaches, for whom patients’ narratives and their transformation are crucial.

Furthermore, systemic therapists, by virtue of their theory and training, should resort to triadic inference fields in explaining symptomatic behaviors of their clients, but with a few exceptions (Coulehan et al., 1998), this topic has been left unexplored and therefore deserves further in-depth investigation. As is well known (Bowen, 1976; Najavits, 1997; Spurling, 2018), therapeutic practice is not always consistent with theory, an eventuality that systemic therapists, like other therapists, cannot avoid (Wolpert, 2000).

No less interesting is the etiopathogenetic content of the explanations provided by clients and therapists. For years now, the scientific debate has tended to put etiopathogenesis of symptoms in brackets. Moreover, the mainstream of family therapy has placed a sort of *epoché* on this subject, a choice motivated by the difficulty in collecting empirical evidence capable of demonstrating precise links between family dynamics and psychopathological disorders, and by attempting to de-pathologize mental health issues, avoiding diagnostic labels. Also, the hope to gain better family engagement, avoiding the potential blame that some families feel in the search of the links between symptoms and family dynamics, played a role. As a result, for several years now, the role of family dynamics in the development of psychological disorders has been somewhat disregarding, and some family therapists claimed that the participation of the family does not imply that they play any role in the etiology of psychopathology (Dare and Eisler, 1997; Lock et al., 2001). These therapists seem to extend to family therapy what Cerletti said about electric shock: it works, but we don’t know why. However, we can assume that patients continue to ask questions about the origin of their symptoms, a subject that concerns them too closely to be evaded. But what kind of explanations for their symptoms do clients propose? Do they prefer interpersonal explanations, as the choice of a systemic therapist would suggest, or do they prefer intrapersonal explanations, as some studies (Stratton et al., 1990; Wolpert and March, 1995; March and Harris, 1996; Wolpert, 2000; Stratton, 2003a, b; Tompkins et al., 2016) suggest?

## Aims and Hypotheses

This study explores the inference fields and the type of etiopathogenetic contents of the client’s and therapist’s explanations, provided during the first two sessions of systemic psychotherapy. At least partially, symptoms and symptomatic behaviors slip away from intentional control, giving back a self-perception in which people often struggle to recognize themselves. Therefore, these behaviors are the most enigmatic an individual can experience, and consequently, they may spur on attributive effort and complexity of the explanations.

A preliminary aim of our study is to explore who, between therapist and client, provides a larger number of explanations about symptoms and symptomatic behaviors.

A second aim, central to our study, is to verify if clients, when trying to give meaning to their symptoms along with their therapists, used almost exclusively monadic and dyadic explanations, very rarely resorting to explanations able to embrace three actors or more, like the university students in Ugazio et al. (2012).

A third aim is to test if, even in the first sessions, the systemic therapist made more use of triadic explanatory schemes than her clients, as her model suggests.

Finally, our study aims to analyze the content of the explanations about symptoms provided by the client and therapist, in order to verify if client and therapist differ in the type of etiopathogenetic content of their explanations, as found in other studies (Wolpert and March, 1995; Wolpert, 2000).

The hypotheses of the study are:

*Hypothesis 1*. Did clients and therapists contribute to a different extent in providing symptom explanations? We expect that clients had introduced more explanations than the therapist.

*Hypothesis 2.* Did monadic and dyadic explanations prevail over triadic explanations during the therapeutic conversation? We expect that both clients and the therapist had utilized more monadic and dyadic explanations than triadic explanations.

*Hypothesis 3.* Did clients and therapists differ in producing triadic explanations? We expect that the therapist had utilized more triadic explanations than her clients.

*Hypothesis 4.* Did clients and therapists provide different types of etiopathogenetic explanations about symptoms? We expect that the therapist had utilized more interpersonal explanations than their clients.

## Materials and Methods

### Participants

This study was carried out on the first two sessions (*M_*time*_* = 1 h 21 min; time range: 1 h to 1 h 37 min; *SD* = 11 min 40 s) of 25 video-recorded and transcribed individual systemic therapies, conducted by the first author in a private institute.^7^

The purpose of the first two sessions, preliminary to a possible psychotherapy, is to understand and contextualize the client’s problems, to examine their current relational situation and family history, as well as to negotiate the effective possibilities of the treatment and its format (individual, couple, or family sessions) (Ugazio, 1984, 1985a, 1985b, 1989).

The clients presented phobic, obsessive–compulsive, eating, or depressive disorders, which met the criteria for a full diagnosis according the *DSM-5* (American Psychiatric Association, 2013). None of them presented psychotic symptoms or satisfied the criteria for a psychotic disorder.^8^

All clients were adults (*M_*age*_* = 38.25 years; age range: 21–59; *SD* = 12.41), both genders (11 males and 14 females), with a high educational level (16 university graduates and 9 high-school graduates), and except for one 20-year-old university student, all had steady work. The sessions were individual and conducted according to the systemic approach. Family members were not involved, because they were unavailable or explicitly requested not to be included by the client.

### Coding and Classifying Procedure

The coding and classifying system “1 to 3: from the monad to the triad” (Ugazio et al., 2008) was applied to the transcripts. It allows to detect the symptom explanations provided by both the conversational partners and to classify them according to breadth of inference field. Adopting a contextual approach, this coding system identifies an explanation as a minimum text unit with an explicative meaning or as a causal chain, that is, as two or more minimum text units linked one to another through the same pattern of semantic coherence. It implies two coding phases: (a) unitizing and (b) reassembling. In the first, the minimum text units are detected; in the second phase, the minimum text units are reassembled and classified as a single explanation if they have the same pattern of semantic coherence.

The detected explanations are classified according to the inference field, using five categories, and operationalized as follows. The examples provided come from the present data corpus.

1. *Monadic.* The symptom explanation is sought within the individual.

Example:

Cl.: “I build up, build up, … I don’t know what to call it… stress? I’d burdened myself with too many responsibilities and in the end, I couldn’t bear them anymore, because I am too emotional…”

2. *Unidirectional dyadic.* The symptom explanation involves two characters, only one of which has an active influence on the other.

Example:

Cl.: “I suffered from anorexia for many years. I recently overcame it, … I think it was because of my mother, she has always wanted me to be perfect, she has always criticized me for every small flaw.”

3. *Bidirectional dyadic.* The symptom explanation entails two characters, both of them are actively involved.

Example:

Cl.: “I felt bad because he was improving, he was becoming better and better and I wasn’t able to keep up with him.”

4. *Triadic*. The symptom explanation involves three or more characters but only partially links them.

Example:

Cl.: “When my sister found out she had Crohn’s disease she drove herself crazy… and my husband had just come back from the hospital and he wanted to be served… I’ve carried the weight of these two sick people on my shoulders… I felt tremendously guilty… then boom! Vomiting has always been my way of reacting, and look at me!”

5. *“Systemic” triadic.* The symptom explanation involves three or more actors, linking them in a circular gestalt.

Example:

Th.: “without this thick layer of fat how would it be possible for you to stay at home? You have the will to live… you are energetic, talkative, you could also be very attractive… then again you feel that your mother is alone, unable to involve your dad, who is still very involved with his family of origin”

The content of each explanation was coded in five categories, three of which corresponded with those used by Schweizer et al. (2010).

1. *Traumas and external events.* Symptoms are attributed to events, which the client considers traumatic or constructs as external. Such events could also be positive, nonetheless, clients believe they have no control over them.

Examples:

Cl.: “A promotion was forthcoming and it caused me very intense anxiety.”

Cl.: “I don’t know, I believe that my panic attacks stem from the violence that I underwent when I was 8 years old. I can’t remember but there was a trial. And then there is a curious fact… when I was doing mental visualizations, I kept seeing the window of a garage… but I didn’t understand why. I talked to my mother and she told me that, as a matter of fact, I was raped in a garage.”

2. *Biomedical explanations.* Symptoms are attributed to genetic or hereditary factors, to organic diseases, or to physiological dysfunctions of the client.

Examples:

Cl.: “Even my grandmother had panic attacks. She never wanted to leave her home. Sometimes I managed to convince her to go out. My mother says it’s a hereditary thing, I agree with her.”

Cl.: “In our family depression has a long history…. My brother killed himself because he was afraid of sinking into depression again. Also, my dad committed suicide. In any case, his mother – my grandmother who I resemble- had always been depressed. So, it’s not so hard to understand why I am depressed. So far all the physicians I consulted have the same opinion.”

3. *Personality traits.* Symptoms are attributed to stable personality traits.

Examples:

Cl.: “I am the most insecure and hesitant person in the world. My father has always said: ‘You are afraid of everything.’ And it’s true, I’ve always been like that! Before I suffered from gastritis, and now I have this kind of thing.”

Cl.: “I am a black sheep, like my grandfather. I got my temper from my father, who is a rebel like me. But they are males, and in my house, there is space for a male black sheep, but not for a female one. Indeed, they are fine, whereas I’ve been diagnosed with bipolar disorder.”

4. *Intrapsychic conflicts.* Symptoms are attributed to dilemmas or conflicts within the person.

Examples:

Cl.: “They told me that all these controls (compulsive behavior) stem from guilty feelings. Actually, they started with the sexual problem. At the time of University, I began to have strong desires and guilty feelings connected to masturbation.”

Cl.: “I don’t know why I’ve always had these (panic) attacks. I don’t know, maybe it is because I like to wander alone, I would also like to travel alone but then I’m scared. I am always fighting between the desire to do, go, see and the fear that something will happen to me.”

5. *Interpersonal conflicts.* Symptoms are attributed to interpersonal conflicts or difficulties.

Examples:

Cl.: “I’ve really suffered due to the envy and the jealousy I had toward my sister. She has always been thin as a twig. I wanted to imitate her but all of my diets were failures, so I began to vomit.”

Cl.: “I feel like I’m choking, I get anxious, especially with my husband, you know, as happens in all relationships, there are these tangles… which make me (miming to choke herself). Yes, I feel a tightness in my stomach and I get tremendously anxious.”

A coder identified and coded the inference fields of all the explanations provided during the 50 sessions. A second coder analyzed the explanations detected in 25% of all the sessions (n = 200). For the inference fields, the inter-rater agreement is 0.77, while for the etiopathogenetic explanations. Cohen’s kappa is higher (0.86).

### Data Analysis

The participants produced 744 explanations during the 50 analyzed sessions, on average, 14.94 per session (range: 12–71). The data were analyzed:

• By frequencies of units in the coding categories and interactions between categories
• By case (client and therapist) as an observation unit

The study design includes the following variables: speaker (client–therapist), inference field, and content. In order to obtain an adequate frequency in each cell, the inference field variable was collapsed into its three main levels: monadic, dyadic, and triadic. Also, the content variable was clustered in three main categories: external causes (trauma and external events), intrapersonal characteristics (biomedical explanations, personality traits, intrapsychic conflicts), and interpersonal dilemma and conflicts.

A log-linear analysis Speaker (2) × Inference field (3) × Content(3) was carried out on the data set of all the explanations provided by clients and therapist (*N* = 744).

Yates’s chi-squared test and *z*-scores were performed on each couple (*N* = 25) and of both individuals (*N* = 25). In order to compare the client’s and therapist’s explanatory contributions using the analysis by each case, a factor model Inference field × Speaker was carried out on the detected explanations. Furthermore, two variables were devised: the most frequent inference field (mfIF) and the broadest inference field (bIF).

## Results

### Did Clients Provide More Explanations Than the Therapist?

As hypothesized, the clients were the main players of the symptom explanations during the two sessions. They provided 71% of total explanations detected during the therapeutic conversation of the first two sessions (529 vs. 215).

The analysis by case shows that each client provided, on average, 21.28 explanations of his or her symptoms (*s* = 8.48), whereas the therapist, with each client, provided, on average, 8.60 (*s* = 8.33) explanations of his or her symptoms.

The difference is significant, even if using an alternative one-tailed hypothesis: *t*(24) = 6.58, *p* < 0.001.

The first hypothesis thus received full confirmation with both kinds of analyses (by frequencies and by case).

### Did Explanations With a Narrower Inference Field Prevail Over Explanations With a Broader Inference Field During the Therapeutic Conversation?

The frequency analysis (*N* = 744) shows that the monadic explanations were the most frequent (50.54%), the unidirectional dyadic were in second place (35.22%), followed by the bidirectional dyadic (8.46%), and finally – with a considerable gap – the triadic explanations (5.78%) followed. Therefore, the trend was ordinal, regardless of whether we take into account five or three levels.

The log-linear analysis Speaker × Inference field × Content shows the significance of the main effect inference field: the differences between each level of the breadth of the inference field are significant (see Table 1).

**TABLE 1:** Frequencies, percentages, and standardized parameters of significant effects resulting from the log-linear standardized parameter analysis (*N* = 744).

		*N*						Standardized parameters			
Effect		Content						Content			
A		External	106	14.25%				External	−3.801***		
CONTENT		Intrapersonal	251	33.74%				Intrapersonal	–2.128		
		Interpersonal	387	52.01%				Interpersonal	8.749***		
		Total	744	100%							
		**Inference field**						**Inference field**			
B		Monadic	376	50.54%				Monadic	9.743***		
INFERENCE FIELD		Dyadic	325	43.68%				Dyadic	3.168**		
		Triadic	43	5.78%				Triadic	−7.203***		
		Total	744	100%							
C		**Speaker**						**Speaker**			
SPEAKER		Clients	529	71.1022%				Clients	3.201**		
		Therapist	215	28.8978%				Therapist	−3.201**		
		Total	744	100%							
		**Inference field**						**Inference field**				
AB			**Monadic**	**Dyadic**	**Triadic**	**Total**			**Monadic**	**Dyadic**	**Triadic**
CONTENT × INFERENCE FIELD	CONTENT	External	82	23	1	106	CONTENT	External	2.059	–0.755	–0.667
		Intrapersonal	228	22	1	251		Intrapersonal	5.683***	–2.558	–1.527
		Interpersonal	66	280	41	387		Interpersonal	−10.803***	4.789***	3.266**
		Total	376	325	43	744					
		**Speaker**						**Speaker**				
AC			**Clients**	**Therapist**	**Total**				**Clients**	**Therapist**	
CONTENT × SPEAKER	CONTENT	External	84	22	106		CONTENT	External	1.403	–1.403	
		Intrapersonal	199	52	251			Intrapersonal	1.949	–1.949	
		Interpersonal	246	141	387			Interpersonal	−3.410**	3.410**	
		Total	529	215	744						
BC		**Speaker**						**Speaker**				
INFERENCE FIELD × SPEAKER			**Clients**	**Therapist**	**Total**				**Clients**	**Therapist**	
	INFERENCE FIELD	Monadic	285	91	376		INFERENCE FIELD	Monadic	2.755*	−2.755*	
		Dyadic	236	89	325			Dyadic	5.510***	−5.510***	
		Triadic	8	35	43			Triadic	−5.493***	5.493***	
		Total	529	215	744						

*p < 0.05; **p < 0.01; ***p < 0.001 with Bonferroni.

The analysis by case highlights a similar trend. The most frequently used inference field by each client–therapist couple (*N* = 25) was the monadic one: being the most used for 15 out of 25 couples, and on average, each couple used 15 monadic explanations (*M* = 15.04). The dyadic one followed: for 10 out of 25 couples, it was the most frequent inference field. On average, each couple used 13 dyadic explanations. None of the couples resorted mainly to the triadic inference field, and on average, each couple used less than two triadic explanations (*M* = 1.72).

Yates’s chi-squared test confirms the significance of the differences between levels: χ^2^(2) = 12.09, *p* < 0.01. Moreover, the monadic inference field was significantly the most used by the client–therapist couple: *z* = 2.31, *p* < 0.05.

These results allow us to affirm that the variable breadth of the inference field identifies levels of increasing complexity in the construction of symptom explanations. It was a trend which seems to follow an economic principle: the most frequent explanations were the least complex, even when the meaning-making involves enigmatic behaviors, such as symptoms.

### Did the Explanations of Symptoms Provided by the Therapist Have a Broader Inference Field Than the Ones Given by Her Clients? Did the Therapist Utilize Triadic Thinking More Than Her Clients?

The log-linear analysis Speaker × Inference field × Content, carried out on the detected explanations produced during the 50 sessions (*N* = 744), allows us to answer affirmatively to this question.

The interaction Speaker × Inference field is significant, as shown in Table 1. These data enable us to identify the direction of the differences between the therapist and the client. Clients utilized more monadic and dyadic explanations than the therapist, and fewer triadic ones. The gap between the therapist and her clients in the triadic explanations was evident: 16.28% of the total explanations versus 1.51%. The systemic explanations provided by the clients were nearly all absent: only one client utilized this kind of explanation, while the systemic explanations were approximately half of all the triadic explanations provided by the therapist.

The log-linear analysis (see Table 1) shows also a significant effect of the interaction Content × Inference field, which is self-evident.

**FIGURE 1:**
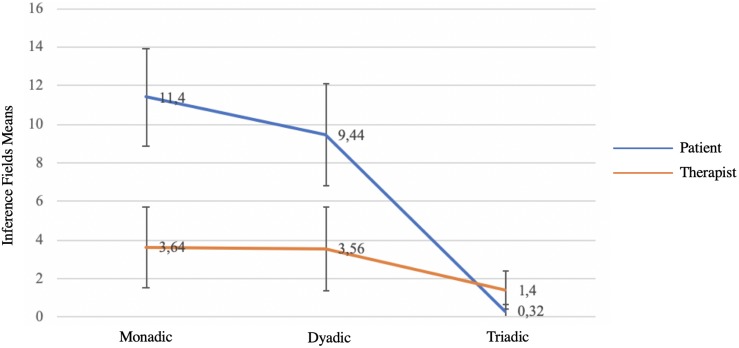
Inference fields used by clients and therapist for the symptom explanations (N = 25).

The factor model Inference field × Speaker, carried out on the explanations given by the 25 client–therapist couples, reveals a significant interaction between the two factors [*F*(2.48) = 14.75, *p* < 0.001; partial η^2^ = 0.38]. The clients’ explanations were nearly exclusively monadic and dyadic, whereas the therapist’s explanations had an almost balanced distribution (see Figure 1).

Furthermore, the following variables were analyzed for each speaker: the mfIF and the bIF. Yates’s chi-squared test, applied to the variable mfIF, only for clients, highlights a significant difference between the three considered levels (monadic, dyadic, and triadic): χ^2^(2) = 12.09, *p* < 0.01, as shown in Table 2. For clients,^9^ the monadic inference field was significantly the most frequent, while the triadic inference field was the least frequent. For the therapist, there were no significant differences for this variable.

**TABLE 2:** Most frequent and broadest inference field used by clients and therapist: frequencies, percentages, and zeta scores (analysis by case, N = 25)

	**Most frequent inference field**		
**Clients**	**Monadic**	**Dyadic**	**Triadic**
*N* (=25)	15	10	0
%	60	40	0
Z	2.31*	0.58	−2.89**
**Therapist**			
*N* (=25)	11	10	4
%	44	40	16
*Z*	0.92	0.58	−1.50
	**Broadest inference field**		
**Clients**	**Monadic**	**Dyadic**	**Triadic**
*N*(=25)	0	20	5
%	0	80	20
**Therapist**			
*N* (=25)	2	8	15
%	8	32	60
*Z*	−1.44	3.42**	−2.89**

*p < 0.05; **p < 0.001.

The application of *z*-scores to the variable bIF shows significant client–therapist differences in two out of the three levels of this variable. The broadest inference field used by the clients was dyadic, while the broadest inference field used by the therapist was triadic (see Table 2).

The third hypothesis is thus also confirmed: the therapist provided more complex explanations, that is, with a broader inference field than the clients. The therapist also utilized the triadic explanatory level to a greater extent than her clients.

### Did the Client’s and Therapist’s Explanations Differ in Terms of Content as Well?

The interpersonal explanations were 50% of all explanations (*n* = 744). The traumatic and the intrapersonal ones were respectively 14.11 and 32.52%, of which the biomedical ones were 6.45%.

The log-linear analysis (see Table 1) shows a significant effect of the interaction Content × Speaker. As the standardized parameter analysis highlights, the therapist utilized fewer traumatic (10.23 vs. 15.87) and intrapersonal (24.18 vs. 37.61) explanations than her clients, whereas the therapist utilized more interpersonal explanations (65.58 vs. 46.50). It is interesting to notice that, beyond these differences, they both show a similar trend in terms of frequency, with the interpersonal explanations in first place, the intrapersonal explanations in second, and lastly, traumatic explanations.

## Discussion and Conclusion

Both the analyses by frequencies and by case confirmed the hypotheses of this study, which explored a variable – the breadth of the inference field of the explanations – not hitherto empirically tested. The exploratory nature of this study is also set by its main limit: the number of participants is rather small, and all the sessions were conducted by the same therapist.

The results show that the clients proved to be the main characters of the attributive plot, while the therapist’s role seemed secondary but not marginal. Even in the early sessions, and in coherence with its technique, the systemic interview was able to restore an agency to the clients that had previously been undermined by their symptoms. Prompted by the therapist’s questions, the clients elaborated on the majority of explanations, hypotheses, and conjectures of their symptoms. The therapist also introduced some explanations, but the responsibility for making sense of their symptoms was mainly taken by their clients. This result is an indirect confirmation that the therapist, in coherence with the systemic technique of conducting the therapeutic session (Selvini Palazzoli et al., 1980; Tomm, 1987,1988), primarily maintained a maieutic position during the sessions, which some systemic therapists have dubbed “a position of *not-knowing*” (Anderson and Goolishian, 1992, p. 28). In this perspective, therapists help their clients to construct, refine, and change their point of view, and consequently their emotions, through their method of questioning rather than assuming an instructive position, which inevitably undermines the client’s agency.

The breadth of the inference field variable was characterized by an ordinal trend: monadic explanations prevailed (50.53), dyadic explanations followed (43.69), and triadic ones were in last place, with a considerable gap between them and the others (5.78). Hence, this variable seems to gauge levels of increasing complexity from the monad to the triad, and triadic thinking resulted uncommon, at least, for the analyzed therapeutic conversation. Each client–therapist couple provided an average of less than two triadic explanations during the first two sessions.

As hypothesized, the explanations put forward by the therapist had a broader inference field than those provided by the client. This finding was confirmed by all the analyses, and it is perhaps the most significant result of this study. The therapist’s attributive trend proved to be different from that of her clients. The therapist also provided fewer triadic explanations than dyadic and monadic ones, but her triadic explanations reached 16.28%, while the clients’ percentage was insignificant (1.51%). Nearly the total of triadic explanations was provided by the therapist, who had already begun to introduce hypotheses and problem redefinitions in the first two sessions, resorting to triadic schemes.

Similarly, support was borne out for the hypothesis that systemic therapists utilized more interpersonal explanations than their clients. A less predictable finding was that the interpersonal explanations were the most frequently used by clients in their narrated story of symptoms and symptomatic behaviors. Clients, at least our participants who had chosen a systemic therapist, proposed relatively few biomedical (6.45%) or traumatic (14.11%) explanations for their symptoms.

These findings allow us to confirm what was supported by the previous study (Ugazio et al., 2012) of which this is a development: that triadic thinking is extraneous, although not unknown, to common sense. Triadic explanations were produced almost exclusively by the therapist. Our results also confirm that the variable breadth of the inference field has an ordinal nature; therefore, it is characterized by levels of increasing complexity.

It is worth mentioning some differences between our results and those of Ugazio et al. (2012) in which all the participants were students. Our clients provided fewer triadic and monadic explanations than the students, respectively 1.51% versus 4.7% and 3.88% versus 60%, but used more dyadic explanations (44.61%, vs. 35.3%). These differences are an additional confirmation that the triadic hermeneutic seems extraneous to common sense. The study’s findings were achieved in a valid ecological context, more reliable than the stimulus situations utilized by Ugazio et al. (2012), where the evoked context was manipulated. Furthermore, these findings arise from conversations between clients and their systemic therapist, who, as we have already highlighted, elicited broad inference fields with an interviewing method based on reflexive and circular questions. These questions widen the conversation to the clients’ entire relational context, as well to positionings of the family members and to family interactive patterns. Nevertheless, even in this context, apart from some exceptions, clients did not access the triadic level. In addition, the majority of explanations with a triadic inference field found in the Ugazio et al. (2012) study had arisen from the triadic enigmatic context, an artificial stimulus situation that tends to force the use of triadic explanations.

The prevalence, for both client and therapist, of interpersonal explanations compared to intrapersonal and traumatic ones implies that, already at the beginning of the therapeutic process, both shared a relational perspective able to favor the therapeutic alliance.

The majority of our participants did not fully embrace psychopathological intrapersonal explanations for disorders (either biomedical or those based on intrapsychic traits and conflicts), which seem so predominant in other studies, at least in the United States (Lebowitz and Appelbaum, 2019). This contrasting trend may reflect an important contextual difference: in Italy, direct-to-consumer advertising for psychotropic drugs is still not allowed, while in the United States, these commercials have very successfully broadcast biochemical explanations of mental disorders and a related conception of disorders as an individual problem. However, even in our sample, the client’s and therapist’s positions were distant because the therapist significantly used more interpersonal explanations than her clients.

We can conclude that our exploratory study, showing that clients and therapists used different inference fields, offers a validation of a premise of one of the most accepted ideas of change in systemic therapies. This is the idea that people change when, thanks to triadic thinking, they gain a new perspective on their past, symptoms, and positions in the contexts they belong to (Sluzki, 1992; Coulehan et al., 1998). According to this perspective, a crucial component of the therapeutic change is precisely the dissonance between client and therapist regarding the inference fields utilized. Thanks to this dissonance, clients and therapists can create a new story, potentially able to change the clients’ feelings, without disconfirming their emotions (Burk et al., 1998; Heatherington et al., 2005). Our study is not able to support this perspective of change, as this was not among our aims. Its design limits the analysis to the inference fields of the first two sessions; furthermore, it did not explore the clients’ feedback to the therapist’s explanations. According to its objectives, our study supported a fundamental premise on which this perspective is based, that client and therapist adopt different inference fields.

Although clear and in coherence with a previous study (Ugazio et al., 2012), our findings cannot be generalized, because of the small number of participants. However, this study provides the instruments to make it replicable. Future investigations should increase the number of participants. Moreover, a comparison between sessions conducted by different therapists, also with different clinical models, could prove of great interest. The comparison between the first sessions and the subsequent ones could allow verifying if clients of systemic therapists widen their inference field, embracing the triadic hermeneutics introduced by their therapists. Lastly, how the client and the therapist co-construct triadic explanations during the sessions could be a captivating topic for qualitative studies coherent with a constructionist perspective.

## Data Availability Statement

The datasets generated for this study are available on request to the corresponding author.

## Ethics Statement

Ethical review and approval was not required for the study on human participants in accordance with the local legislation and institutional requirements. The participants provided their written informed consent to participate in this study.

## Author Contributions

VU developed the hypotheses and the design of the study, and wrote the manuscript with the collaboration of SG. RP coded the 80% of the data as first coder. LF coded the 20% of the data as first coder and the 25% of the data as second coder. RP, LF, and SG found the references. PA performed the statistical analyses. All authors approved the submitted version of the manuscript and agreed to be accountable for all aspects of the work.

## Conflict of Interest

The authors declare that the research was conducted in the absence of any commercial or financial relationships that could be construed as a potential conflict of interest. The handling editor declared a shared affiliation with one of the authors PA at the time of review.

## Footnotes

1 The therapist is the first author of this study.

2 The therapist, who is the first author of this study, as mentioned in the previous note, already had more information prior to suggesting this hypothesis. In any case, according to systemic method and practice, this hypothesis, as all the other ones, is no more than a supposition, useful in suggesting questions with a broad field of inference (Selvini Palazzoli et al., 1980). Answering the therapist’s questions, clients develop, confirm, invalidate, or introduce a new hypothesis different from the suggested one, thereby expressing their agency threatened by the onset of the disorder.

3 “Sara/Marco is a model student attending Medicine and needs to pass only five more exams before graduating. From one day to the next she/he decide to pull out of University.”

4 “Sara/Marco is a model student attending Medicine and needs to pass only five more exams before graduating. From one day to the next she/he decide to pull out of University and tell her/his mother, who burst into tears.”

5 “Sara/Marco is a model student attending Medicine and needs to pass only five more exams before graduating. From one day to the next she/he decide to pull out of University and tell her/his parents: her/his mother burst into tears and her/his father tries to comfort his wife.”

6 “Sara/Marco is a model student attending Medicine and needs to pass only five more exams before graduating. From one day to the next she/he decide to pull out of University and tell her/his parents: her/his mother burst into tears, while her/his father seems pleased.”

7 The therapist is Valeria Ugazio, who carried out the psychotherapies at the European Institute of Systemic-relational Therapies (www.eist.it), a private institute based in Milan (Italy), where the video-recordings are stored.

8 The first clinical group is composed of nine clients affected by phobic disorders: two of them with agoraphobia, without a clinical history of panic disorder (F40.00), three with panic disorder and agoraphobia (F40.00 and F41.0), and four with panic disorder without agoraphobia (F41.0). The second group is formed by six clients affected by obsessive–compulsive disorder (F42). The third one is composed of eight clients affected by eating disorders: four with anorexia nervosa (F50.01 and F50.02), and four with bulimia nervosa (F50.2). The last group is formed by two female clients affected by a major depressive disorder (F33.2).

9 Of the explanations, 3.36% were classified in a residual category which gathers explanations with different types of contents or adopted when the coders were uncertain about coding.
